# Establishment of prognostic nomogram in cervical cancer with hepatitis B virus infection: a retrospective study

**DOI:** 10.3389/fonc.2026.1826692

**Published:** 2026-07-13

**Authors:** Zhongyan Dou, Shuhui Yu, Jinping Zhang, Xingrao Wu, Kangming Li, Meiping Jiang, Chunfang Zhao, Lan Zhang

**Affiliations:** 1 Department of Radiation Oncology, The Third Affiliated Hospital of Kunming Medical University, Yunnan Cancer Hospital, Peking University Cancer Hospital Yunnan, Kunming, China; 2 Department of Medical Administration, The Third Affiliated Hospital of Kunming Medical University, Yunnan Cancer Hospital, Peking University Cancer Hospital Yunnan, Kunming, China

**Keywords:** antiviral therapy, cervical cancer, HBsAg-positive, nomogram, prognosis

## Abstract

**Background:**

This study aimed to identify prognostic factors and establish a nomogram for predicting overall survival (OS) in hepatitis B surface antigen-positive (HBsAg-positive) cervical cancer (CC) patients.

**Methods:**

A retrospective analysis of 149 HBsAg-positive CC patients treated at Yunnan Cancer Hospital (2015–2017) was performed. Cox regression identified independent prognostic factors for nomogram development. The nomogram’s predictive accuracy and discriminative capability were evaluated through Harrell’s concordance index (C-index), calibration plots, and decision curve analysis, and its performance was compared to the International Federation of Gynecology and Obstetrics (FIGO) staging system. Internal validation was conducted using the bootstrap resampling method.

**Results:**

Multivariate Cox regression analyses identified tumor stage, baseline serum AST levels, antiviral therapy, lymph node status, and treatment modality as independent prognostic factors for patients with HBsAg-positive CC, all of which were incorporated into the nomogram for OS. The Harrell’s C-index of nomogram was 0.817 (95% confidence interval [CI], 0.762–0.873), which was higher than that of FIGO staging system 0.700 (95% CI, 0.637–0.764, *P* < 0.001). Moreover, the nomogram showed superior performance in decision curve analysis (DCA) and a higher area under the curve (AUC) compared to the FIGO staging system. The calibration curve for survival probability exhibited good consistency between the probabilities and observed values. Grouping based on the nomogram’s cutoff value showed that the high-risk group experienced significantly poorer OS than the low-risk group (*P* < 0.001).

**Conclusions:**

This study established a nomogram for the preliminary prognostic evaluation of OS in HBsAg-positive CC patients. Further validation is required prior to its clinical application.

## Introduction

Cervical cancer (CC) constitutes the most common gynecologic malignancy worldwide, accounting for an estimated 604,000 incident cases and 342,000 deaths in 2020. In China, there are 106,000 newly occurred cases and 48,000 death reports every year (1). Chronic hepatitis B virus (HBV) infection remains a substantial public health burden in China, and is a major cause of chronic hepatitis, cirrhosis, and hepatocellular carcinoma (HCC) (2, 3). Recent evidence indicated an association between HBV and multiple extrahepatic malignancies, such as gastric cancer, endometrial carcinoma, and CC (4–10). Cancer patients with chronic HBV infection, who are considered hepatitis B surface antigen-positive (HBsAg-positive), have an inferior overall survival (OS), though the underlying mechanism needs further investigation (11–13). Therefore, identifying prognostic factors in patients with HBsAg-positive cancer is crucial for the selection of more individualized treatment strategies.

The International Federation of Gynecology and Obstetrics (FIGO) staging system is now a widely accepted tool for the therapeutic and prognostic administration of CC. The FIGO staging systems divides CC patients into different stages according to anatomical extent such as tumor size, vagina or parametrial involvement, regional lymph node metastasis and distant metastasis. Given the HBV-infected cancer patients are biological heterogenous population, a more comprehensive prognostic predictor considering clinical and laboratory information is necessary for them. Variable liver function indicators (LFIs), including alanine aminotransferase (ALT), aspartate aminotransferase (AST), ALT-to-AST ratio (LSR), AST-to-ALT ratio (SLR), gamma-glutamyl transpeptidase (GGT), lactate dehydrogenase (LDH), alkaline phosphatase (ALP), total bilirubin (TBIL), total bile acid (TBA), and albumin (ALB), have been noticed prognostic values in cancer patients with or without HBV infection (14–21). Patients with chronic HBV infection are at risk of HBV reactivation during chemotherapy. Provision of antiviral therapy for HBV has the potential to improve the prognosis of nasopharyngeal carcinoma (NPC) patients with concomitant HBV infection (22). To date, no studies have investigated the influence of anti-HBV treatment and LEIs on the prognosis of patients with HBsAg-positive CC.

A nomogram represents a valuable instrument that leverages readily accessible clinical data to furnish straightforward prognostic information for oncologists. Studies have demonstrated that nomograms offer superior predictive accuracy compared to conventional staging systems employed in various malignancies (23, 24). As of yet, no study has developed a prognostic nomogram for HBsAg-positive CC patients. Therefore, in the current study, we aimed to establish a nomogram based on clinical characteristics, anti-HBV treatment and LEIs in hopes of elucidating further prognostic insights for patients with HBsAg-positive CC. We also tested whether the nomogram model provided a more accurate prediction of patient survival in comparison to the FIGO staging system.

## Materials and methods

### Ethics statement

This retrospective study received approval from the Research Ethical Committees of the Third Affiliated Hospital of Kunming Medical University (Kunming, China) (approval number: KYLX2022142) and was conducted in adherence to the ethical principles outlined in the Declaration of Helsinki. Informed consent was dispensed with by the committee on account of the retrospective nature of this study. The confidentiality of patient information was preserved by excluding personally identifiable information from the analytical process.

### Patient selection

This retrospective observational study included 149 CC patients with chronic HBV infection who initially presented to the Third Affiliated Hospital of Kunming Medical University (Kunming, China) between January 2015 and December 2017. The inclusion criteria were as follows (1): tumors were histologically confirmed cervical squamous carcinoma, adenocarcinoma, or adenosquamous carcinoma; (2) patients with HBsAg-positive, but without evidence of co-infection with other types of hepatitis viruses; (3) patient without human immunodeficiency virus (HIV) infection; (4) patient without a second tumor; (5) patients had comprehensive baseline clinical information, and active follow-up was conducted with complete records of follow-up dates; (6) patients with Karnofsky ≥ 80 score.

### Clinical variables extracted for analysis

Baseline clinicopathologic parameters of the patients, including age, stage, histological type, tumor differentiation, maximal tumor size, LFIs, HBV serum markers, lymph node metastasis status, HPV status, HBV deoxyribonucleic acid (DNA) level and treatment history were obtained from the electronic medical records system. LFIs including ALT, AST, LSR, SLR, LDH, GGT, TBA, ALP, ALB and TBIL. HBV serum markers including HbsAg, hepatitis B surface antibody (HbsAb), hepatitis B e antigen (HbeAg), hepatitis B e antibody (HbeAb) and hepatitis B core antibody (HbcAb) were recorded. Neoplasm staging was established by consensus of two experienced gynecologic oncologists utilizing data obtained from vaginal and bimanual pelvic examination, computed tomography (CT) of the chest and abdomen with iodine-containing contrast medium, and magnetic resonance imaging (MRI) of the pelvis, or positron emission tomography-CT (PET-CT), adhering to the FIGO 2009 cervical cancer criteria. Pathological diagnosis was based on the comprehensive cervical control guidelines of the World Health Organization (WHO). In patients who did not undergo pelvic lymph node dissection, lymph nodes showing a short-axis diameter > 10 mm, specific apparent diffusion coefficient (ADC) values on imaging, or ring enhancement and marked necrosis on imaging were defined as metastatic.

### Hepatitis and HBV reactivation definition

In our hospital, the upper limit of normal of serum ALT level is 40 IU/L. Hepatitis was defined as a greater than 3-fold increase in serum ALT levels exceeding 100 IU/L. In this study, we assessed whether hepatitis occurred by monitoring patients’ baseline ALT levels upon admission and before and after any treatment. HBV reactivation was defined as either an increase exceeding 10-fold from baseline levels or the attainment of an absolute HBV DNA level greater than 10^9^ IU/mL during the treatment period and within the first year following treatment completion (25).

### Serum LFIs, HBV, HPV, HIV and other hepatitis viruses assay

Liver function tests were performed on all patients upon admission. The serum LFIs was repeated weekly or biweekly during hospitalization. Serum LFIs were measured using a Roche Cobas 8000 automatic biochemical analyzer (Roche Diagnostics GmbH, Mannheim, Germany), and the relevant test kits were purchased from Roche Diagnostics. Quality control procedures for the measurements were executed in accordance with the manufacturer’s protocols. Rate method was used to detect ALT, AST, LDH, ALP, GGT. The values of TBIL were measured based on the chemical oxidation method. Serum ALB was measured using the bromocresol green method. Measurement of serum TBA by using the enzymatic cycling method.

Routine HBV screening was conducted on all patients at their initial visit. Serum was separated from whole blood specimens and employed for HBV infection testing. Several HBV-related antibodies and antigens were tested, including HbsAg, HbsAb, HbeAg, HbeAb and HbcAb. Enzyme-linked immunosorbent assay (ELISA) (WANTAI BioPharm, Beijing, China) was applied for this test. We assigned specific personnel the responsibility of ensuring the prompt delivery of blood specimens for laboratory analysis to maintain the validity of the results. Cut-off values for the different hepatitis B markers were defined using the mean values of manufacturer-provided negative control samples for each commercial kit. Quality control for hepatitis B marker determination adhered to the manufacturer’s guidelines. In brief, the assay results for all positive and negative quality control samples were required to be accurately categorized as specified in the instructions for each kit. Deviant results from quality control samples necessitated repeat analysis. Additionally, reference positive and negative control serum from the Ministry of Health of the People’s Republic of China was incorporated into this testing procedure to assure accuracy. In most cases, the tests were performed for qualitative detection. HBV DNA was not routinely detected. HBV DNA levels were quantified by real-time polymerase chain reaction, with a lower limit of detection of 100 IU/mL.

HPV testing included the detection of viral DNA and mRNA (E6/E7) using nucleic acid hybridization and polymerase chain reaction, following standard laboratory protocols. Blood specimens were also tested for antibodies to HIV, hepatitis A virus (HAV), hepatitis C virus (HCV), hepatitis D virus (HDV), and hepatitis E virus (HEV), and HDV antigens using ELISA.

### Treatment

In this study, patients were categorized into 4 groups based on the primary treatment, including (1) radical surgery alone (RSA); (2) definitive chemoradiotherapy or radical radiotherapy alone (DCRT/RRA); (3) radical surgery combined with chemoradiotherapy or radical surgery combined with radiotherapy/chemotherapy (RS-CRT/RS-R/C); and (4) other treatments. Of these, 20 patients were in the RSA group, 54 in the DCRT/RRA group, 45 in the RS-CRT/RS-R/C group, and 30 in the other treatments group. The other treatments include chemotherapy alone and symptomatic supportive treatment without anticancer therapy.

Radical surgery consisted of type C hysterectomy and pelvic lymph node dissection (external iliac nodes, internal iliac nodes, common iliac nodes, suprainguinal nodes, parametrial nodes, and obturator nodes) with or without paraaortic lymphadenectomy.

Radiotherapy comprises external beam radiation and brachytherapy. The radiotherapeutic modalities included two-dimensional radiotherapy, three-dimensional conformal radiotherapy, intensity-modulated radiotherapy, and volumetric-modulated arc therapy. The gross tumor volume (GTV) comprised the cervix, the uterus, and the neoplastic lesion. The clinical target volume (CTV) encompassed the GTV with a 5- to 10-mm expansion margin and included the parametrium, upper vagina, and pelvic lymph node regions (common iliac, external iliac, internal iliac, and presacral regions) (26). The GTVnd incorporated metastatic lymph nodes. The CTV was expanded by a 5–10 mm margin in all directions to delineate the planning target volume (PTV). The planning gross tumor volume (PGTVnd) was generated by the addition of a 5-mm margin to the GTVnd. A prescribed total radiation dose of 40–50.4 Gy was delivered to the PCTV in 25–28 daily fractions. Concurrently, the PGTVnd received a boost to a dose of 55–60 Gy. High dose rate intracavity brachytherapy, with a total dose of 20–30 Gy delivered in four to five fractions, was to be initiated at week five of external beam radiotherapy.

For patients receiving concurrent chemoradiotherapy, the concurrent chemotherapeutic regimen consisted of either weekly cisplatin administered at a dosage of 30–40 mg/m² or paclitaxel (175 mg/m²) in combination with cisplatin (75 mg/m²). The neoadjuvant/adjuvant chemotherapy and chemotherapy alone also consisted of paclitaxel (175 mg/m^2^) plus cisplatin (75 mg/m^2^). A subset of patients presenting with renal insufficiency were administered carboplatin in lieu of cisplatin, with a dose area under the curve of 5.

### Antiviral treatment

We retrospectively reviewed the electronic medical record system and identified 24 patients who received antiviral therapy among 149 HBsAg-positive patients with cervical cancer. Of these 24 patients, 3 were newly diagnosed with hepatitis B after admission and received antiviral therapy for at least one week prior to the initiation of any pre-treatment. The remaining 21 patients had a prior diagnosis of chronic hepatitis B and had been on continuous antiviral treatment. All patients receiving antiviral therapy were administered entecavir 0.5 mg tablets orally once daily. The antiviral therapy was continued for at least six months after the last treatment. The other 125 patients without antiviral therapy included both patients with prior hepatitis B diagnosis and those newly diagnosed after admission. We speculate that the decision to administer or withhold antiviral therapy in these patients resulted from a combination of factors, including their physical conditions, personal preferences, physicians’ clinical judgment, and disparities in medical resources at that time.

### Follow-ups and endpoints

Follow-up for patient survival data was conducted through the retrieval of medical records or by means of direct telephone communication. The follow-up of all patients continued until the occurrence of death or May 23, 2022. The endpoint of this study was OS, defined as the time from the date of diagnosis to the date of death due to any cause, or until the last follow-up.

### Statistical analyses

Statistical analyses were performed using SPSS 26.0 software (IBM, Chicago, IL, USA) and the nomograms were performed in R software (version 4.2.1). The Kaplan-Meier survival plots were performed with GraphPad Prism [version 9.3.0 (463)]. X-tile 3.6.1 (Yale University, New Haven, CT, USA) was used to determine the optimal cut-off value for ALB, TBIL, LDH, GGT, TBA, AST, LSR, and SLR levels. Categorical variables were categorized according to clinical findings, and compared using chi-square tests or Fisher’s exact tests. The baseline demographic and clinical characteristics are presented as percentages or as median values with ranges. Cox proportional hazards regression analysis was performed to determine the hazard ratio (HR) and corresponding 95% confidence interval (CI) for factors associated with OS. Baseline parameters were initially subjected to univariate Cox proportional hazards regression analysis to evaluate their association with OS. Those variables with a statistical significance of *P* < 0.10 were subsequently included in a multivariate Cox proportional hazards regression model. Backward stepwise selection with the Akaike Information Criterion was applied in multivariate Cox regression model to test the independent significance of different factors, and retained marginally significant variables (*P* < 0.05) in the final Cox model. The derived multivariate Cox regression model was employed for the calculation of a risk score and the construction of the final prognostic nomogram. Utilizing the identified predictive factors for OS derived from the final model, a nomogram was developed to predict the 1-, 3-, and 5-year OS probabilities for CC patients with HBsAg-positive. The Harrell’s concordance index (C-index) ranging between 0.5 and 1.0 and time-dependent receiver operating characteristic (t-ROC) curves were used to evaluate the nomogram. The predictive effect of the nomogram was internally verified by the calibration curve through bootstrap sampling 1,000 times. Decision curve analysis (DCA) was employed to evaluate the clinical performance and net benefit of the nomogram. Based on the total scores of nomogram model, patients were separated into either low or high-risk subgroups using the X-tile software. Subsequently, the log-rank tests and Kaplan-Meier analyses were performed between the high-risk and low-risk group to evaluate the predictive ability of the prognostic nomogram. *P* values less than 0.05 were considered statistically significant.

## Results

### Patient clinical characteristics

In total, 149 eligible patients with serologically positive for HBsAg CC were met the study inclusion criteria. Patients’ demographic and clinicopathologic characteristics are presented in **Table 1**; **Supplementary Table 1**. As for the distribution of age, we categorized patients into two groups: ≤ 50 years and >50 years. The number of patients classified into these two groups were 90 (60.4%) and 59 (39.6%), respectively. The average age of patients was 49.0 ± 9.8 years, ranging from 27 to 75 years (median age of 49 years). The majority of patients were classified as FIGO stage II disease (45.6%), approximately 85.2% of patients had squamous carcinoma. There were 4 patients (2.7%) who were seropositive for HBsAb, 19 patients (12.8%) were seropositive for HBeAg, 116 patients (77.9%) seropositive for HBeAb, and 148 patients (99.3%) seropositive for HBcAb. 9 patients (6.0%) examined the serum HBV DNA at diagnosis, while 6 patients (4.0%) were examined during treatment, and no patient was found to experience reactivation of HBV DNA. Before treatment, 2 patients in the antiviral therapy group had hepatitis and 3 patients in the non-antiviral therapy group had hepatitis. During treatment, only 6 patients in the non-antiviral therapy group had hepatitis. And no patient was identified as experiencing HBV DNA reactivation. In all, only 24 (16.1%) patients received antiviral therapy. The median follow-up time was 63.0 months (range:0.4–90.0 months).

**Table 1:** Baseline clinical characteristics in 149 HBsAg-positive cervical cancer patients.

Variables (n = 149)	
FIGO staging system, n (%)	
I	50 (33.6)
II	68 (45.6)
III	27 (18.1)
IV	4 (2.7)
Lymph nodes metastases, n (%)	
No	94 (63.1)
Yes	55 (36.9)
Antiviral therapy, n (%)	
No	125 (83.9)
Yes	24 (16.1)
baseline serum AST levels (U/L), n (%)	
≤40	133 (89.3)
>40	16 (10.7)
Treatment modality, n (%)	
RSA	20 (13.4)
DCRT/RRA	54 (36.2)
RS-CRT/RS-R/C	45 (30.2)
other treatments	30 (20.1)

FIGO, International Federation of Gynecology and Obstetrics; AST, aspartate aminotransferase; RSA, radical surgery alone; DCRT/RRA, definitive chemoradiotherapy or radical radiotherapy alone; RS-CRT/RS-R/C, radical surgery combined with chemoradiotherapy or radical surgery combined with radiotherapy/chemotherapy.

As presented in **Supplementary Tables 2**, **7** of the 11 patients with hepatitis were attributed to being in the active phase. Other possible causes of hepatitis included concurrent chemoradiotherapy (1patient), chemotherapy (1 patient), and infection (2 patients). The median value of the ALT levels during hepatitis was 146 U/L (range, 123–231 U/L). In total, no hepatitis-related mortalities were observed, including deaths caused by acute hepatic failure, upper gastrointestinal bleeding, cirrhosis, or hepatocellular carcinoma (27, 28).

### Comparison of baseline characteristics between the antiviral therapy group and the non-antiviral therapy group

Baseline characteristics of patients with and without antiviral therapy are shown in **Supplementary Table 3**. No significant differences were observed between the two groups regarding age (*p* = 0.495), FIGO stage system (*p* = 0.803), histology (*p* = 0.871), histological differentiation (*p* = 0.468), maximal tumor size (*p* = 0.219), lymph nodes metastases (*p* = 0.391), liver-protective drugs (*p* = 0.545), fatty liver (*p* = 0.933), liver cirrhosis (*p* = 1.000), use of corticosteroids (*p* = 0.173), treatment modality (*p* = 0.438), and baseline serum ALB (*p* = 0.711), LDH (*p* = 0.385), ALP (*p* = 0.516), GGT (*p* = 0.739), TBA (*p* = 0.200), TBIL (*p* = 0.266), AST (*p* = 0.724), SLR (*p* = 0.512), LSR (*p* = 0.293), hepatitis activity (*p* = 0.691), HBV DNA reactivation (*p* = 0.469), HBeAg (*p* = 1.000), HBeAb (*p* = 0.366) and HBcAb (*p* = 1.000) status. Significant differences were observed in HPV status (*p* = 0.018) and HBsAb status (*p* = 0.013) between the two groups. Further analysis of HPV status found significant disparities in HPV-positive and unknown status between groups (*p* = 0.014). Specifically, the antiviral therapy group had a significantly higher proportion of HPV-positive cases and a lower proportion of cases with unknown HPV status compared with the non-antiviral group. Regarding the between-group difference in HBsAb status, we speculate that sustained viral suppression after antiviral treatment stimulated the synthesis of protective HBsAb, which led to this intergroup discrepancy.

Due to some patients having unknown data regarding HBV DNA reactivation and HPV status, these two indicators were excluded from the Cox proportional hazards regression model. In addition, this cohort included patients with pre-existing hepatitis B and those newly diagnosed with hepatitis B after admission. Since HBV serological markers (HBsAb, HBeAg, HBeAb and HBcAb) may undergo seroconversion after antiviral therapy, these markers were also excluded from the model to eliminate potential confounding bias.

### Sifting independent prognostic factors

Before constructing the nomogram model, we included a total of 22 demographic and clinical variables in univariate Cox analysis and multivariate Cox analysis to identify independent predictors. In the univariate analysis, tumor stage, maximal tumor size, cirrhosis, baseline serum ALB, TBIL, LDH, ALP, GGT, TBA, AST levels, SLR, antiviral therapy, lymph node status and treatment modality were significantly associated with OS. The *P*-values of these variables were all less than 0.1. Following stepwise selection to further remove potential redundancy, we identified five variables with *P* < 0.05, which were tumor stage, baseline serum AST levels, antiviral therapy, lymph node status and treatment modality. **Table 2** and **Supplementary Table 4** showed the details of univariate and multivariate analysis results.

**Table 2:** Univariate and multivariate analyses for overall survival of 149 HBsAg-positive cervical cancer patients.

Variable	Univariate analysis for OS		Multivariate analysis for OS	
	HR (95% CI)	*P*-value	HR (95% CI)	*P*-value
FIGO staging system		<0.001		<0.001
I	1(Reference)		1(Reference)	
II	3.697(1.521-8.986)		3.623(1.426-9.201)	
III	8.834(3.496-22.325)		10.362(3.590-29.908)	
IV	12.264(3.052-49.284)		13.922(3.227-60.052)	
Lymph nodes metastases		<0.001		0.001
No	1(Reference)		1(Reference)	
Yes	3.208(1.855-5.548)		2.754(1.550-4.894)	
baseline serum AST levels (U/L)		0.086		0.042
≤40	1(Reference)		1(Reference)	
>40	1.934(0.911-4.108)		2.290(1.029-5.097)	
Antiviral therapy		0.039		0.017
No	1(Reference)		1(Reference)	
Yes	0.342(0.123-0.948)		0.280(0.098-0.799)	
Treatment modality		<0.001		<0.001
RSA	1(Reference)		1(Reference)	
DCRT/RRA	5.131(1.209-21.769)		1.129 (0.244-5.231)	
RS-CRT/RS-R/C	1.344(0.271-6.661)		0.806(0.160-4.050)	
other treatments	13.038(3.055-55.652)		4.911(1.100-21.922)	

HR, hazard ratio; 95% CI, 95% confidence interval; FIGO, International Federation of Gynecology and Obstetrics; AST, aspartate aminotransferase; RSA, radical surgery alone; DCRT/RRA, definitive chemoradiotherapy or radical radiotherapy alone; RS-CRT/RS-R/C, radical surgery combined with chemoradiotherapy or radical surgery combined with radiotherapy/chemotherapy; OS, overall survival.

*P* value was calculated using a Cox proportional hazards model.

Values in bold correspond to data showing significance.

Based on these independent prognostic factors, we analyzed the associations between each predictor and OS. As presented in **Supplementary Table 5**, a total of 96 patients were alive and 53 patients had died by the date of the last follow-up in our cohort. Stratified analyses by tumor stage revealed 6 deaths in stage I, 26 deaths in stage II, 18 deaths in stage III, and 3 deaths in stage IV. There were 49 deaths among patients without antiviral therapy and 4 deaths among those receiving antiviral therapy. Thirty-one deaths occurred in patients with lymph nodes metastases, compared with 22 deaths in patients without lymph nodes metastases. Forty-five deaths were observed in patients with baseline serum AST ≤40 U/L, and 8 deaths were recorded in those with baseline serum AST >40 U/L. In terms of treatment modalities, 2 deaths occurred in patients receiving RSA, 23 deaths in patients receiving DCRT/RRA, 6 deaths in patients receiving RS-CRT/RS-R/C, and 22 deaths in patients receiving other treatments. Significant intergroup differences were identified between survivors and non-survivors with respect to tumor stage (*p* < 0.001), antiviral therapy status (*p* = 0.001), lymph node metastasis (*p* < 0.001), and treatment modality (*p* < 0.001). Although baseline serum AST levels showed no statistically significant difference between the two groups (*p* = 0.202), the proportion of patients with baseline serum AST >40 U/L was higher among deceased patients, whereas the proportion of patients with baseline serum AST ≤40 U/L was lower in the mortality group relative to surviving patients.

### Development and verification of prognostic nomogram

**Figure 1:**
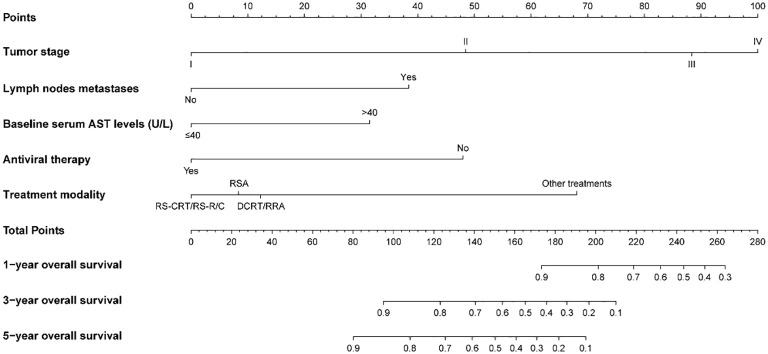
Nomogram predicting the 1-, 3- and 5-year overall survival (OS) in HBsAg-positive CC patients. The nomogram was utilized to summate the point values identified on the point scale for each variable. The aggregate point score, as projected onto the bottom scales, indicates the probability of 1-, 3-, and 5-year survival, respectively. AST, aspartate aminotransferase; RSA, radical surgery alone; DCRT/RRA, definitive chemoradiotherapy or radical radiotherapy alone; RS-CRT/RS-R/C, radical surgery combined with chemoradiotherapy or radical surgery combined with radiotherapy/chemotherapy.

Based on the results from the multivariable Cox proportional hazards regression models, we developed nomogram incorporating the independent prognostic factors to predict 1-, 3-, and 5-year OS (**Figure 1**). By aggregating the scores associated with each variable and subsequently projecting the total score onto the respective scales, the estimated probabilities of 1-, 3-, and 5-year OS can be readily calculated. With respect to each patient, a higher score was deemed predictive of a less favorable prognosis.

**Figure 2:**
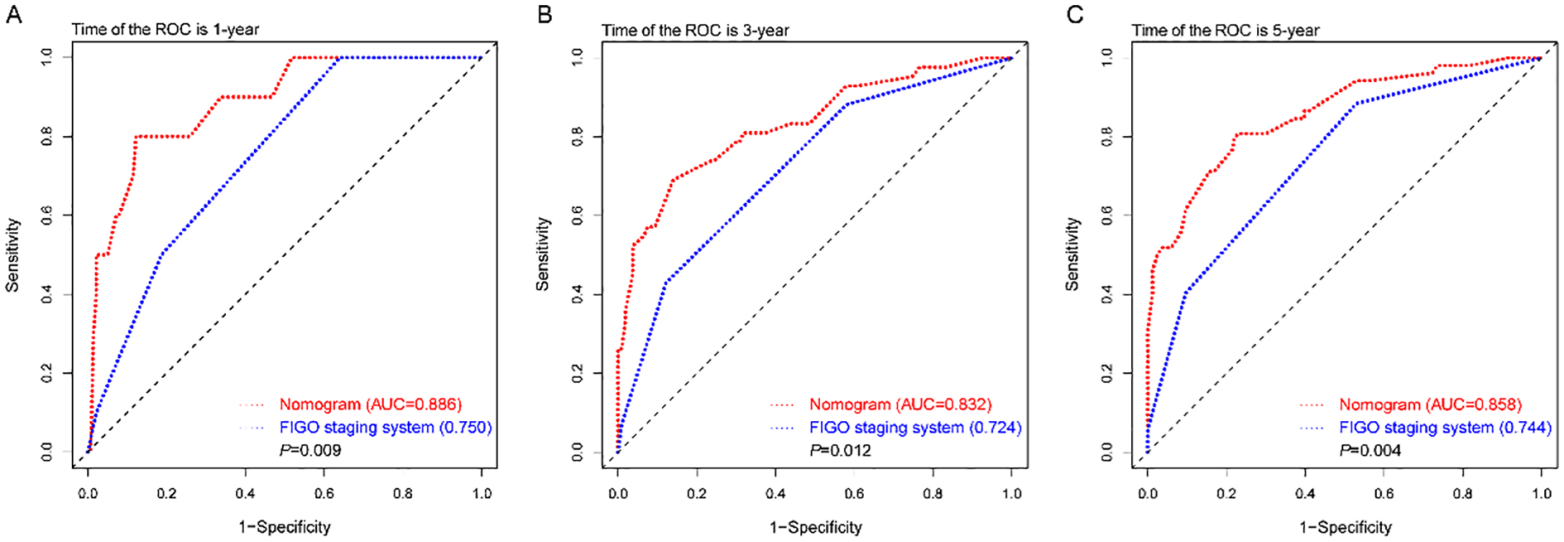
Time-dependent operating characteristics curves (t-ROC) for predicting patient overall survival (OS) at **(A)** 1 year, **(B)** 3 years and **(C)** 5 years. The red dashed lines represent the nomogram prediction of OS. The blue dashed lines depict the FIGO staging system prediction of OS. AUC: area under the curve.

Validation of the nomogram encompassed assessments of discrimination and calibration. Discrimination was evaluated using the Harrell’s C-indexes and t-ROC curves. The Harrell’s C-indexes were used to evaluate the predictive accuracies of the nomogram model and the conventional FIGO staging system. A C-index value of 1 is indicative of perfect discriminative ability, while a value of 0.5 indicates a random guess. For the nomogram model, the C-index was 0.817 (95% CI, 0.762–0.873), which indicated that the model had better discriminatory ability that of the FIGO staging system, with a value of 0.700 (95% CI, 0.637–0.764, *P* < 0.001). The t-ROC curve serves as a valuable tool for characterizing the sensitivity and specificity of predictive models. The ROC area under the curve (AUC) values for the nomograms predicting 1-, 3-, and 5-year OS were 0.886, 0.832, and 0.858, respectively. While the 1-, 3-, and 5-year AUC values of the FIGO staging system for OS were 0.750, 0.724 and 0.744, respectively (**Figure 2**). The AUC values of the 1-, 3- and 5-year OS nomograms were obviously higher than those of the FIGO staging system (*P* = 0.009, *P* = 0.012, *P* = 0.004, respectively).

**Figure 3:**
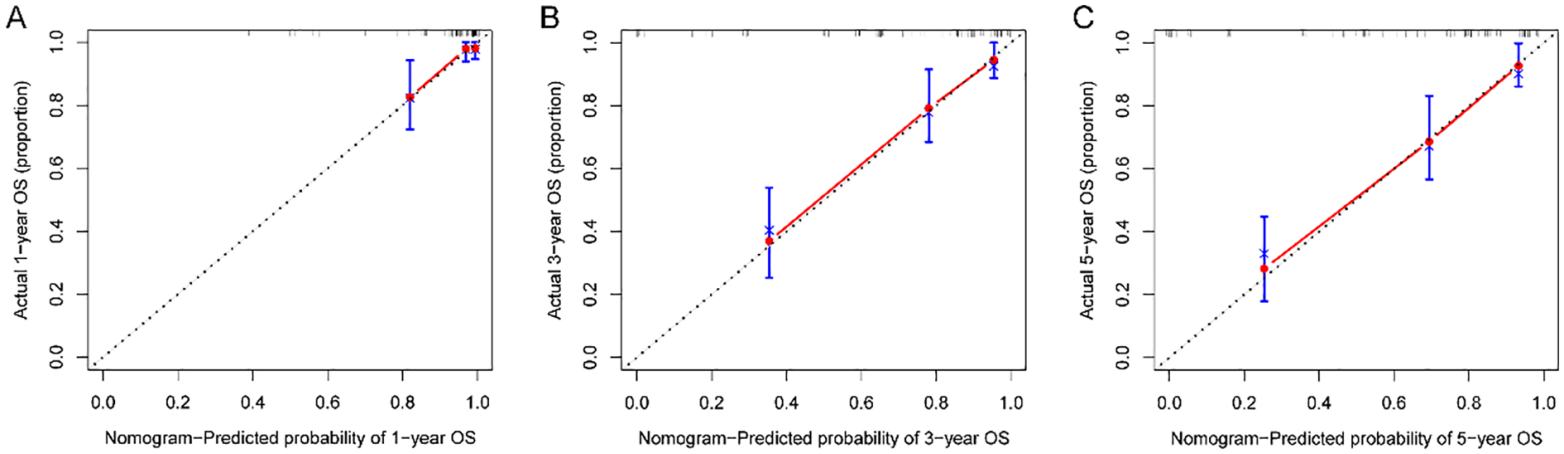
Calibration curves of the nomogram for predicting HBsAg-positive cervical cancer (CC) patient overall survival (OS) at **(A)** 1 year, **(B)** 3 years and **(C)** 5 years. The nomogram model graphically represented predicted OS along the abscissa and observed OS along the ordinate. The solid red line represents the performance of the nomogram model; closer alignment with the diagonal black dashed line indicates improved estimation.

Calibration enables the assessment of the disparity between predictions and observed outcomes, and effectively illustrates the relationship between true probability and predicted probability. As depicted in **Figure 3**, the calibration curves, with no significant deviations from the reference line, indicated optimal concordance between model predictions and actual observed outcomes for 1-, 3-, and 5-year OS.

**Figure 4:**
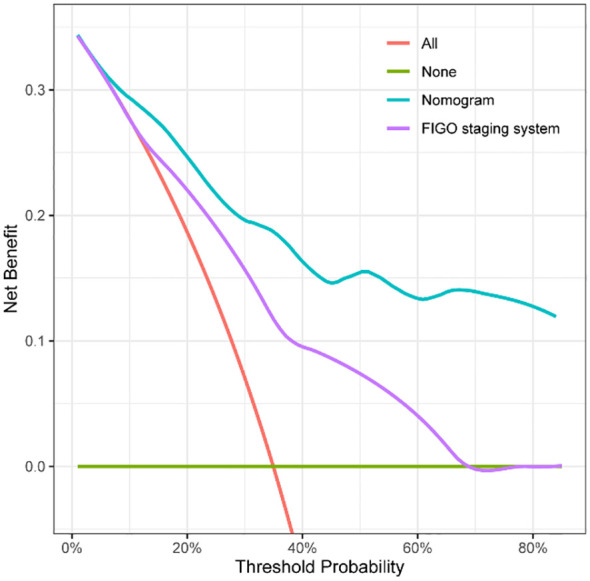
Decision curve analysis of 5-year survival prognosis. In decision curve analysis, the ordinate represents the net benefit, which is calculated by aggregating the benefits (true positives) and subtracting the harms (false positives). Green line: all patients dead. Red line: none patients dead. Blue line: model of nomogram. Purple line: model of FIGO staging system. FIGO, International Federation of Gynecology and Obstetrics.

DCA for the nomogram and FIGO staging systems was showed in **Figure 4**. The DCA indicated that the developed nomogram and FIGO staging system exhibited superior performance in predicting OS compared to scenarios of universal mortality or universal survival when the probability threshold for a patient was > 10%. By using DCA to ascertain the clinical usefulness of the nomograms, it was found compared with FIGO staging system, nomogram showed superior net benefit with a wider range of threshold probability and improved performance for predicting OS. This suggested that the nomogram could more accurately predict the prognosis of HBsAg-positive CC patients.

### Performance of the nomogram model in the stratification of patient risk

**Figure 5:**
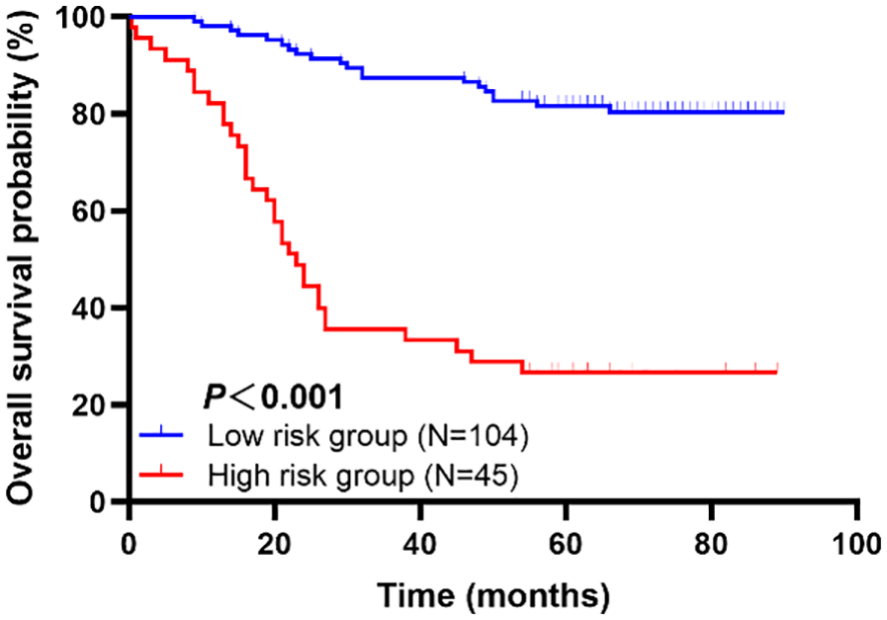
Kaplan-Meier survival curves of nomogram stratified by X-tile software. Overall survival was significantly shorter in the high-risk group than in the low-risk group.

As per the optimal cut-off values calculated by X-tile software, we grouped all participants into low-risk or high-risk group based on the predictions of the nomogram model. The high-risk group contained 45 patients, while the low-risk group contained 104 patients. OS of patients in these two groups was evaluated via Kaplan-Meier analysis. The results showed that patients in high-risk group had shorter OS (32.9 ± 25.3 months) than those in low-risk group (65.3 ± 19.6 months, *P* < 0.001), indicating a significant unfavorable outcome for high-risk HBsAg-positive CC patients (**Figure 5**).

## Discussion

The prevalence of HBV infection is a major health problem worldwide. Among all countries, China is one of the regions with a relatively high incidence rate (29). The long-term survival outcome of HBsAg-positive CC patients remain poorly understood and they were often ruled out by most of the clinical trials. Traditional FIGO staging system considers only the anatomical extent of the disease without taking into account the liver bio-functional heterogeneity and application of anti-HBV treatment of HBsAg-positive CC, which cannot fully reflect the accurate prognosis. Given the limitations of the FIGO staging system in accurately predicting prognosis for HBsAg-positive CC, the development of robust predictive models for this patient group is clinically imperative. Additionally, previous study has found that HBV antigens are present in CC tumor tissue and suggested that HBV infection is associated with CC (8). Therefore, it is important to consider the influence of HBV in HBsAg-positive CC.

In this study, we evaluated the prognostic ability of anti-HBV treatment and LEIs in HBsAg-positive CC patients to establish a preliminary predictive nomogram model for them by combining anti-HBV treatment and LEIs with clinicopathological features. To our knowledge, this is the first preliminary nomogram model for the prognosis of HBsAg-positive CC patients. We found that tumor stage, baseline serum AST levels, antiviral therapy, lymph node status and treatment modality were independent predictors of patient prognosis as per the multivariate analysis. Patients with an earlier tumor stage, a lower baseline serum AST level, the used of antiviral treatment, without lymph nodes metastases, and RS-CRT/RS-R/C therapeutic have improved survival rates. In comparison to the conventional FIGO staging system, our model demonstrates superior accuracy in predicting patient survival by incorporating anatomical factors, antiviral therapy, and baseline hepatic biochemical parameters, and exhibits a higher concordance rate in patients with HBsAg-positive CC. The nomogram model also showed a higher overall net benefit than the FIGO staging system at 5 years.

HBV infection remains a major chronic infectious disease burden in humans. Intrahepatic persistence and active replication of HBV are the main causes of hepatic dysfunction, chronic hepatitis, cirrhosis, other HBV-related liver diseases, and the development of HCC. Timely and effective antiviral therapy can suppress HBV replication and reactivation, ease hepatic inflammation, and improve hepatic function reserve, which is the most effective measure to delay and prevent progression or exacerbation of the disease, and it can alter the natural history of chronic hepatitis B (CHB) (30). The current therapeutic armamentarium for CHB comprises interferon-alpha (Peg-IFNα) and nucleos(t)ide analogs (NAs), specifically nucleosides such as lamivudine, telbivudine, and entecavir, and nucleotides such as adefovir and tenofovir. These agents effectively suppress HBV replication and prevent novel viral integrations. The fundamental objective in the therapeutic approach to CHB is the achievement of maximal suppression of HBV replication. IFN is typically of limited duration, whereas NAs are generally prescribed for extended periods, often requiring lifelong administration (31–33). NAs are characterized by a favorable safety profile, are generally well-tolerated, and can effectively suppress viral replication to undetectable levels. Attenuation of hepatocellular inflammation and necrosis, hepatic fibrosis, and hyperplasia can be achieved through the inhibition of HBV replication, consequently delaying and reducing the occurrence of severe complications such as hepatic failure, cirrhosis, and HCC (34, 35). Ample studies have shown that antiviral therapy improves the prognosis of HBV-related HCC patients (17, 36, 37). In contrast, there are relatively few reports on the effects of antiviral therapy on extrahepatic tumors. A study of combined antiviral therapy in NPC patients receiving chemotherapy showed that lamivudine reduced the incidence of HBV reactivation and chemotherapy-related hepatitis (38). Weng et al. compared the 5-year distant metastasis-free survival (DMFS) rates of HBV-infected NPC patients in the antiviral group with those in the non-antiviral group, and found that the 5-year DMFS of the antiviral group was significantly higher than that of the non-antiviral group, suggesting that anti-hepatitis B treatment may improve the long-term prognosis of patients with HBV-infected NPC (22). Meta-analyses showed that prophylactic antiviral therapy with lamivudine decreased the incidence of HBV-related hepatitis, incidence of HBV reactivation and rate of chemotherapy disruption compared with HBsAg-positive breast cancer patients without antiviral therapy (39, 40). Prophylactic lamivudine administered during chemotherapy in HBsAg-positive breast cancer patients may positively influence treatment outcomes. Researches concerning other malignancies have also indicated antiviral therapy can decrease the incidence of HBV reactivation (41, 42). Our study also found that antiviral therapy was an independent prognostic factor related to OS in HBsAg-positive CC, and patients who received antiviral therapy had a superior long-term prognosis. We consider that HBsAg-positive CC patients receiving antiviral therapy tend to obtain superior hepatic function reserve than patients without antiviral therapy, which may decrease the probability of drug induce liver injury. It also decreases the probability of chemotherapy-associated HBV reactivation and avoids interruption of the treatment process. In this regard, adherence to antiviral therapy is indispensable for achieving and maintaining viral suppression, leading to long-term improvement in liver function. Therefore, our findings suggest that HBsAg-positive CC patients should receive antiviral therapy as early as possible, in order to allow a better chance of clinical benefit.

In addition to antiviral therapy, LFIs are also a potential prognostic factor that should not be neglected, which can intuitively reflect the hepatic function reserve and extent of liver injury of patients. Numerous studies have shown that liver function parameters correlate with prognosis of intra- and extra-hepatic malignancies (43–46). The LEIs included in our study included serum liver enzymes (AST, ALT, LDH, GGT, and ALP), bilirubin (TBIL), bile acids (TBA), and ALB, which provided a comprehensive assessment of liver function in HBsAg-positive CC patients from the dimensions of biosynthesis, uptake, and secretion. Of these, AST is an important hepatocyte enzyme, located mainly in the mitochondria of hepatocytes, which will be released from damaged hepatic cells into the bloodstream after hepatocellular injury or death, and is valuable in the assessment of liver injury (47). AST can be generated not only by hepatocytes but also by malignant tumor cells, and serum AST levels have been found to correlate with the prognosis of various malignancies, including HCC (48), non-small cell lung cancer (14), breast cancer (49), multiple myeloma (50), and, and renal cell carcinoma (19). We identified serum AST level as an independent prognostic factor in the survival of HBsAg-positive CC patients by multivariate Cox regression analyses. Patients with higher serum AST levels tended to have a worse prognosis. We consider that patients with higher baseline serum AST levels were likely to experience liver dysfunction or liver injury prior to anti-tumor therapy, resulting in insufficient liver function reserve, which in turn delayed or interrupted the course of treatment and led to a poor prognosis for the patients. Therefore, a comprehensive and holistic prediction of survival in patients with HBsAg-positive CC should incorporate the baseline serum AST level as a prognostic indicator in patients and not be limited to clinical-anatomical factors.

Clinical prediction models are employed to probe relationships between future clinical outcomes and baseline health status of patients with specific conditions. These models enable the integration of the results of traditional analyses, visualization in nomograms, simplification of the results through more intuitive and convincing presentations, and prediction of the probability of occurrence of certain outcome events by means of a scoring system. In our study, we proposed incorporating multiple potential prognostic factors, including antiviral therapy, baseline serum AST levels, tumor stage, lymph node status and treatment modality, to build a preliminary exploratory clinical prediction model for predicting the prognosis of HBsAg-positive CC patients. This is undoubtedly groundbreaking for the standardized diagnosis and treatment of HBsAg-positive CC patients, and provides personalized medical recommendations for future treatments that are more precise and efficacious.

With these strengths aside, there were several limitations in the development of our nomogram. Firstly, owing to its retrospective design and the exclusion of cases with incomplete data, this study was inevitably subject to selection and recall bias. Despite the implementation of multivariate analysis in this study, bias coexisted with confounding factors, which were difficult to eliminate fundamentally through statistical optimization. The retrospective nature of this study should be taken into account when interpreting these results. Secondly, the nomogram was created only based on data from a single center in a hepatitis B virus-endemic area in China, with a limited sample size. In addition, the absence of an independent validation cohort carries an inherent risk of overfitting and consequently overoptimistic predictive performance. Thus, external validation of the nomogram model necessitates additional multi-center studies with larger sample sizes. In particular, large-scale, multi-center cohort studies conducted across diverse geographic and ethnic groups are essential to verify the universal applicability of our findings. Finally, we only analyzed the effects of biochemical indicators of liver function and antiviral therapy on the prognosis of HBsAg-positive CC patients. Other prognostic factors, such as serum tumor markers, HBV DNA and inflammatory indicators were not included. Therefore, this model cannot be applied to CC patients with other hepatitis or other viral infections. To further improve the model’s predictive ability, future validation studies should encompass additional risk factors. Notwithstanding these limitations, the nomogram model was effective and may be instrumental in predicting the prognosis of HBsAg-positive CC patients.

## Conclusions

In conclusion, we have established a nomogram model to reliably predict the 1-, 3- and 5-year cancer-specific OS in HBsAg-positive CC patients, of which the prognostic value was better than that of the traditional FIGO staging system. This nomogram represents a preliminary exploratory tool for survival prediction in HBsAg-positive CC patients. Further external validation and larger prospective studies are warranted.

## Data Availability

The original contributions presented in the study are included in the article/**Supplementary Material**. Further inquiries can be directed to the corresponding author.

## Glossary

CC: cervical cancer
HBV: hepatitis B virus
HCC: hepatocellular carcinoma
HBsAg-positive: hepatitis B surface antigen-positive
OS: overall survival
FIGO: International Federation of Gynecology and Obstetrics
LFIs: liver function indicators
ALT: alanine aminotransferase
AST: aspartate aminotransferase
GGT: gamma-glutamyl transpeptidase
LDH: lactate dehydrogenase
ALP: alkaline phosphatase
TBIL: total bilirubin
TBA: total bile acid
ALB: albumin
NPC: nasopharyngeal carcinoma
HIV: human immunodeficiency virus
DNA: deoxyribonucleic acid
LSR: ALT-to-AST ratio
SLR: AST-to-ALT ratio
HbsAb: hepatitis B surface antibody
HbeAg: hepatitis B e antigen
HbeAb: hepatitis B e antibody
HbcAb: hepatitis B core antibody
CT: computed tomography
MRI: magnetic resonance imaging
PET-CT: positron emission tomography-CT
WHO: World Health Organization
ADC: apparent diffusion coefficient
ELISA: enzyme-linked immunosorbent assay
HAV: hepatitis A virus
HCV: hepatitis C virus
HDV: hepatitis D virus
HEV: hepatitis E virus
RSA: radical surgery alone
DCRT/RRA: definitive chemoradiotherapy or radical radiotherapy alone
RS-CRT/RS-R/C: radical surgery combined with chemoradiotherapy or radical surgery combined with radiotherapy/chemotherapy
GTV: gross tumor volume
CTV: clinical target volume
ptv: planning target volume
HR: hazard ratio
CI: confidence interval
C-index: concordance index
t-ROC: time-dependent receiver operating characteristic
DCA: decision curve analysis
AUC: area under the curve
CHB: chronic hepatitis B
Peg-IFNα: interferon-alpha
NAs: nucleos(t)ide analogs
DMFS: distant metastasis-free survival
