# Supplementary material for: Establishment of prognostic nomogram in cervical cancer with hepatitis B virus infection: a retrospective study
Source: Front Oncol. 2026 Jul 13;16:1826692. doi: 10.3389/fonc.2026.1826692 (PMC13402118; doi:10.3389/fonc.2026.1826692)
Supplement: Supplementary file 1 [file Table1.docx]

| **Supplementary Table 1** Baseline clinical characteristics in 149 HBsAg-positive cervical cancer patients | |
| --- | --- |
| Variables (n = 149) | |
| Histology, n (%) |  |
| Squamous | 127 (85.2) |
| Adenocarcinoma | 18 (12.1) |
| Adenosquamous | 4 (2.7) |
| Histological differentiation, n (%) |  |
| G1 well-differentiated | 18 (12.1) |
| G2 moderately-differentiated | 87 (58.4) |
| G3 poorly- differentiated | 18 (12.1) |
| Unknown | 26 (17.4) |
| Age (year), n (%) |  |
| ≤50 | 90 (60.4) |
| ＞50 | 59 (39.6) |
| Maximal tumor size (cm), n (%) |  |
| ≤4 | 76 (51.0) |
| ＞4 | 73 (49.0) |
| HPV status, n (%) |  |
| Negative | 22 (14.8) |
| Positive | 95 (63.8) |
| Unknown | 32 (21.5) |
| Liver-protective drugs, n (%) |  |
| Yes | 104 (69.8) |
| No | 45 (30.2) |
| Liver cirrhosis, n (%) |  |
| Present | 3 (2.0%) |
| Absent | 146 (98.0) |
| Fatty liver, n (%) |  |
| Present | 32 (21.5) |
| Absent | 117 (78.5) |
| HbsAb status, n (%) |  |
| Positive | 4 (2.7) |
| Negative | 145 (97.3) |
| HbeAg status, n (%) |  |
| Positive | 19 (12.8) |
| Negative | 130 (87.2) |
| HbeAb status, n (%) |  |
| Positive | 116 (77.9) |
| Negative | 33 (22.1) |
| HbcAb status, n (%) |  |
| Positive | 148 (99.3) |
| Negative | 1 (0.7) |
| baseline serum ALB levels (g/L), n (%) |  |
| ＞39 | 134 (89.9) |
| ≤39 | 15 (10.1) |
| baseline serum LDH levels (U/L), n (%) |  |
| ≤225 | 138 (92.6) |
| ＞225 | 11 (7.4) |
| baseline serum ALP levels (U/L), n (%) |  |
| ≤59 | 30 (20.1) |
| ＞59 | 119 (79.9) |
| baseline serum GGT levels (U/L), n (%) |  |
| ≤46 | 131 (87.9) |
| ＞46 | 18 (12.1) |
| baseline serum TBA levels (µmol/L), n (%) |  |
| ≤11.3 | 129 (86.6) |
| ＞11.3 | 20 (13.4) |
| baseline serum TBIL levels (µmol/L), n (%) |  |
| ≤4.9 | 15 (10.1) |
| ＞4.9 | 134 (89.9) |
| SLR, n (%) |  |
| ≤1.73 | 130 (87.2) |
| ＞1.73 | 19 (12.8) |
| LSR, n (%) |  |
| ≤0.57 | 16 (10.7) |
| ＞0.57 | 133 (89.3) |
| HBV DNA reactivation, n (%) |  |
| Yes | 0 (0.0) |
| No | 14 (9.4) |
| Unknown | 135 (90.6) |
| Use of corticosteroids, n (%) |  |
| No | 68 (45.6) |
| Yes | 81 (54.4) |
| Hepatitis reactive, n (%) |  |
| Present | 11 (7.4) |
| Absent | 138 (92.6) |
| *Abbreviations*: HPV, human papillomavirus; ALB, albumin; LDH, lactate dehydrogenase; APL, alkaline phosphatase; GGT, gamma-glutamyl transpeptidase; TBA, total bile acid; TBIL, total bilirubin (TBIL); HBV, hepatitis B virus; DNA, deoxyribonucleic acid; LSR, ALT-to-AST ratio (LSR), SLR, AST-to-ALT ratio (SLR); HbsAb, hepatitis B surface antibody; HbeAg, hepatitis B e antigen; HbeAb, hepatitis B e antibody; HbcAb, hepatitis B core antibody. | |

| **Supplementary Table 2** Incidence of Hepatitis, Biochemical Features, and Clinical Outcomes | |  |
| --- | --- | --- |
|  | Patients with hepatitis reactive(n=11) |  |
| Outcome |  |  |
| Incidence of hepatitis, *n (%)* |  |  |
| Hepatitis attributable to patients in the active phase | 7 (63.6) |  |
| Hepatitis attributable to concurrent chemoradiotherapy | 1 (9.1) |  |
| Hepatitis attributable to chemotherapy | 1 (9.1) |  |
| Hepatitis attributable to infection | 2 (18.2) |  |
| Highest ALT level, median (range), U/L | 146.0 (123.0-231.0) |  |
| Hepatitis-related death, *n (%)* | 0 (0.0) |  |
| *Abbreviations*: HBV, hepatitis B virus; ALT, alanine aminotransferase. | |  |

| **Supplementary Table 3** Clinical characteristics of HBsAg-positive cervical cancer patients with and without antiviral therapy | | | |
| --- | --- | --- | --- |
| Characteristics | Without antiviral therapy group (n=125) | With antiviral therapy group (n=24) | *p* |
| Age (year), n (%) |  |  | 0.495 |
| ≤50 | 77 (85.6) | 13 (14.4) |  |
| ＞50 | 48 (81.4) | 11 (18.6) |  |
| FIGO staging system, n (%) |  |  | 0.803 |
| I | 42 (84.0) | 8 (16.0) |  |
| II | 58 (85.3) | 10 (14.7) |  |
| III | 22 (81.5) | 5 (18.5) |  |
| IV | 3 (75.0) | 1 (25.0) |  |
| Histology, n (%) |  |  | 0.871 |
| Squamous | 105 (82.7) | 22 (17.3) |  |
| Adenocarcinoma | 16 (88.9) | 2 (11.1) |  |
| Adenosquamous | 4 (100.0) | 0 (0.0) |  |
| Histological differentiation, n (%) |  |  | 0.468 |
| G1 well-differentiated | 16 (88.9) | 2 (11.1) |  |
| G2 moderately-differentiated | 71 (81.6) | 16 (18.4) |  |
| G3 poorly- differentiated | 14 (77.8) | 4 (22.2) |  |
| Unknown | 24 (92.3) | 2 (7.7) |  |
| Maximal tumor size (cm), n (%) |  |  | 0.219 |
| ≤4 | 61 (80.3) | 15 (19.7) |  |
| >4 | 64 (87.7) | 9 (12.3) |  |
| Lymph nodes metastases, n (%) |  |  | 0.391 |
| Yes | 48 (87.3) | 7 (12.7) |  |
| No | 77 (81.9) | 17 (18.1) |  |
| Liver-protective drugs, n (%) |  |  | 0.545 |
| Yes | 86 (82.7) | 18 (17.3) |  |
| No | 39 (86.7) | 6 (13.3) |  |
| Fatty liver, n (%) |  |  | 0.933 |
| Present | 27 (84.4) | 5 (15.6) |  |
| Absent | 98 (83.8) | 19 (16.2) |  |
| Liver cirrhosis, n (%) |  |  | 1.000 |
| Present | 3 (100.0) | 0 (0.0) |  |
| Absent | 122 (83.6) | 24 (16.4) |  |
| Use of corticosteroids, n (%) |  |  | 0.173 |
| No | 54 (79.4) | 14 (20.6) |  |
| Yes | 71 (87.7) | 10 (12.3) |  |
| Treatment modality, n (%) |  |  | 0.438 |
| RSA | 16 (80.0) | 4 (20.0) |  |
| DCRT/RRA | 44 (81.5) | 10 (18.5) |  |
| RS-CRT/RS-R/C | 37 (82.2) | 8 (17.8) |  |
| other treatments | 28 (93.3) | 2 (6.7) |  |
| baseline serum ALB levels (g/L), n (%) |  |  | 0.711 |
| ＞39 | 113 (84.3) | 21 (15.7) |  |
| ≤39 | 12 (80.0) | 3 (20.0) |  |
| baseline serum LDH levels (U/L), n (%) |  |  | 0.385 |
| ≤225 | 117 (84.8) | 21 (15.2) |  |
| ＞225 | 8 (72.7) | 3 (27.3) |  |
| baseline serum ALP levels (U/L), n (%) |  |  | 0.516 |
| ≤59 | 24 (80.0) | 6 (20.0) |  |
| ＞59 | 101 (84.9) | 18 (15.1) |  |
| baseline serum GGT levels (U/L), n (%) |  |  | 0.739 |
| ≤46 | 109 (83.2) | 22 (16.8) |  |
| ＞46 | 16 (88.9) | 2 (11.1) |  |
| baseline serum TBA levels (µmol/L), n (%) |  |  | 0.200 |
| ≤11.3 | 106 (82.2) | 23 (17.8) |  |
| ＞11.3 | 19 (95.0) | 1 (5.0) |  |
| baseline serum TBIL levels (µmol/L), n (%) |  |  | 0.266 |
| ≤4.9 | 11 (73.3) | 4 (26.7) |  |
| ＞4.9 | 114 (85.1) | 20 (14.9) |  |
| baseline serum AST levels (U/L), n (%) |  |  | 0.724 |
| ≤40 | 112 (84.2) | 21 (15.8) |  |
| ＞40 | 13 (81.3) | 3 (18.8) |  |
| Hepatitis reactive, n (%) |  |  | 0.691 |
| Present | 9 (81.8) | 2 (18.2) |  |
| Absent | 116 (84.1) | 22 (15.9) |  |
| SLR, n (%) |  |  | 0.512 |
| ≤1.73 | 110 (84.6) | 20 (15.4) |  |
| ＞1.73 | 15 (78.9) | 4 (21.1) |  |
| LSR, n (%) |  |  | 0.293 |
| ≤0.57 | 12 (75.0) | 4 (25.0) |  |
| ＞0.57 | 113 (85.0) | 20 (15.0) |  |
| HPV status, n (%) |  |  | 0.018 |
| Negative | 20 (90.9) | 2 (9.1) |  |
| Positive | 74 (77.9) | 21 (22.1) |  |
| Unknown | 31 (96.9) | 1 (3.1) |  |
| HBV DNA reactivation, n (%) |  |  | 0.469 |
| Yes | 0 (0.0) | 0 (0.0) |  |
| No | 13 (92.9) | 1 (7.1) |  |
| Unknown | 112 (83.0) | 23 (17.0) |  |
| HbsAb status, n (%) |  |  | 0.013 |
| Positive | 1 (25.0) | 3 (75.0) |  |
| Negative | 124 (85.5) | 21 (14.5) |  |
| HbeAg status, n (%) |  |  | 1.000 |
| Positive | 16 (84.2) | 3 (15.8) |  |
| Negative | 109 (83.8) | 21 (16.2) |  |
| HbeAb status, n (%) |  |  | 0.366 |
| Positive | 99 (85.3) | 17 (14.7) |  |
| Negative | 26 (78.8) | 7 (21.2) |  |
| HbcAb status, n (%) |  |  | 1.000 |
| Positive | 124 (83.8) | 24 (16.2) |  |
| Negative | 1 (100.0) | 0 (0.0) |  |
| *Abbreviations*: FIGO, International Federation of Gynecology and Obstetrics; RSA, radical surgery alone; DCRT/RRA, definitive chemoradiotherapy or radical radiotherapy alone; RS-CRT/RS-R/C, radical surgery combined with chemoradiotherapy or radical surgery combined with radiotherapy/chemotherapy; HPV, human papillomavirus; ALB, albumin; LDH, lactate dehydrogenase; APL, alkaline phosphatase; GGT, gamma-glutamyl transpeptidase; TBA, total bile acid; TBIL, total bilirubin (TBIL); AST, aspartate aminotransferase; LSR, ALT-to-AST ratio (LSR), SLR, AST-to-ALT ratio (SLR); HPV, human papilloma virus infection; HBV, hepatitis B virus; DNA, deoxyribonucleic acid; HbsAb, hepatitis B surface antibody; HbeAg, hepatitis B e antigen; HbeAb, hepatitis B e antibody; HbcAb, hepatitis B core antibody. | | | |
| *P* value was calculated using the Chi Square test or Fisher’s exact test. | | | |

| **Supplementary Table 4** Univariate and multivariate analyses for overall survival of 149 HBsAg-positive cervical cancer patients. | | | | | |
| --- | --- | --- | --- | --- | --- |
| Variable | Univariate analysis for OS | |  | Multivariate analysis for OS | |
|  | HR (95% CI) | *P*-value |  | HR (95% CI) | *P*-value |
| Histology |  | 0.123 |  |  | NA |
| Squamous | 1(Reference) |  |  | NA |  |
| Adenocarcinoma | 1.157(0.521-2.573) |  |  | NA |  |
| Adenosquamous | 3.407(1.049-11.060) |  |  | NA |  |
| Histological differentiation |  | 0.078 |  |  | NS |
| G1 well-differentiated | 1(Reference) |  |  | NS |  |
| G2 moderately-differentiated | 1.016(0.389-2.653) |  |  | NS |  |
| G3 poorly- differentiated | 1.953(0.655-5.830) |  |  | NS |  |
| Unknown | 2.174(0.782-6.042) |  |  | NS |  |
| Age (year) |  | 0.253 |  |  | NA |
| ≤50 | 1(Reference) |  |  | NA |  |
| ＞50 | 1.370(0.799-2.350) |  |  | NA |  |
| Maximal tumor size (cm) |  | 0.005 |  |  | NS |
| ≤4 | 1(Reference) |  |  | NS |  |
| ＞4 | 2.236(1.275-3.923) |  |  | NS |  |
| baseline serum ALB levels (g/L) | | 0.002 |  |  | NS |
| ＞39 | 1(Reference) |  |  | NS |  |
| ≤39 | 2.960(1.482-5.913) |  |  | NS |  |
| baseline serum LDH levels (U/L) | | 0.004 |  |  | NS |
| ≤225 | 1(Reference) |  |  | NS |  |
| ＞225 | 3.017(1.419-6.414) |  |  | NS |  |
| baseline serum ALP levels (U/L) | | 0.048 |  |  | NS |
| ≤59 | 1(Reference) |  |  | NS |  |
| ＞59 | 2.354(1.006-5.507) |  |  | NS |  |
| baseline serum GGT levels (U/L) | | 0.032 |  |  | NS |
| ≤46 | 1(Reference) |  |  | NS |  |
| ＞46 | 2.122(1.066-4.227) |  |  | NS |  |
| baseline serum TBA levels (µmol/L) | | 0.023 |  |  | NS |
| ≤11.3 | 1(Reference) |  |  | NS |  |
| ＞11.3 | 2.158(1.110-4.195) |  |  | NS |  |
| baseline serum TBIL levels (µmol/L) | | 0.001 |  |  | NS |
| ≤4.9 | 1(Reference) |  |  | NS |  |
| ＞4.9 | 0.335(0.172-0.653) |  |  | NS |  |
| SLR, n (%) |  | 0.082 |  |  | NS |
| ≤1.73 | 1(Reference) |  |  | NS |  |
| ＞1.73 | 1.843(0.926-3.669) |  |  | NS |  |
| LSR, n (%) |  | 0.174 |  |  | NA |
| ＞0.57 | 1(Reference) |  |  | NA |  |
| ≤0.57 | 0.593(0.280-1.259) |  |  | NA |  |
| Liver-protective drugs |  | 0.221 |  |  | NA |
| No | 1(Reference) |  |  | NA |  |
| Yes | 0.704(0.402-1.235) |  |  | NA |  |
| Liver cirrhosis |  | 0.075 |  |  | NS |
| Absent | 1(Reference) |  |  | NS |  |
| Present | 3.625(0.880-14.927) |  |  | NS |  |
| Fatty liver |  | 0.172 |  |  | NA |
| Absent | 1(Reference) |  |  | NA |  |
| Present | 0.592(0.279-1.256) |  |  | NA |  |
| Use of corticosteroids |  | 0.958 |  |  | NA |
| No | 1(Reference) |  |  | NA |  |
| Yes | 1.015(0.591-1.743) |  |  | NA |  |
| Hepatitis |  | 0.435 |  |  | NA |
| No | 1(Reference) |  |  | NA |  |
| Yes | 1.443(0.574-3.627) |  |  | NA |  |
| Abbreviations: HR: hazard ratio; 95% CI: 95% confidence interval; ALB, albumin; LDH, lactate dehydrogenase; APL, alkaline phosphatase; GGT, gamma-glutamyl transpeptidase; TBA, total bile acid; TBIL, total bilirubin (TBIL); LSR, ALT-to-AST ratio (LSR), SLR, AST-to-ALT ratio (SLR); NA, not applicable; NS, not significant; OS, overall survival. | | | | | |
| *P* value was calculated using a Cox proportional hazards model. | | | | | |

| **Supplementary Table 5** Comparison of independent prognostic factors associated with survival status in HBsAg-positive cervical cancer patients at the last follow-up | | | |
| --- | --- | --- | --- |
| Characteristics | Survival (n=96) | death (n=53) | *p* |
| FIGO staging system, n (%) |  |  | ＜ 0.001 |
| I | 44 (88.0) | 6 (12.0) |  |
| II | 42 (61.8) | 26 (38.2) |  |
| III | 9 (33.3) | 18 (66.7) |  |
| IV | 1 (25.0) | 3 (75.0) |  |
| Antiviral therapy, n (%) |  |  | 0.038 |
| No | 76 (60.8) | 49 (39.2) |  |
| Yes | 20 (83.3) | 4 (16.7) |  |
| Lymph nodes metastases, n (%) |  |  | ＜ 0.001 |
| Yes | 24 (43.6) | 31 (56.4) |  |
| No | 72 (76.6) | 22 (23.4) |  |
| baseline serum AST levels (U/L), n (%) |  |  | 0.202 |
| ≤40 | 88 (66.2) | 45(33.8) |  |
| ＞40 | 8 (50.0) | 8 (50.0) |  |
| Treatment modality, n (%) |  |  | ＜ 0.001 |
| RSA | 18 (90.0) | 2 (10.0) |  |
| DCRT/RRA | 31 (57.4) | 23 (42.6) |  |
| RS-CRT/RS-R/C | 39 (86.7) | 6 (13.3) |  |
| other treatments | 8 (26.7) | 22 (73.3) |  |
| *Abbreviations*: FIGO, International Federation of Gynecology and Obstetrics; RSA, radical surgery alone; DCRT/RRA, definitive chemoradiotherapy or radical radiotherapy alone; RS-CRT/RS-R/C, radical surgery combined with chemoradiotherapy or radical surgery combined with radiotherapy/chemotherapy; AST, aspartate aminotransferase. | | | |
| *P* value was calculated using the Chi Square test or Fisher’s exact test. | | | |
